# 1,1′-[2,3,5,6-Tetra­methyl-*p*-phenyl­ene­bis(methyl­eneoxy)]di-1*H*-benzotriazole

**DOI:** 10.1107/S1600536809010782

**Published:** 2009-03-28

**Authors:** B. Ravindran Durai Nayagam, Samuel Robinson Jebas, Caroline Daisy, Dieter Schollmeyer

**Affiliations:** a Department of Chemistry, Popes College, Sawyerpuram 628 251, Tamilnadu, India; b Department of Physics, Karunya University, Karunya Nagar, Coimbatore 641 114, India; c Institut für Organische Chemie, Universität Mainz, Duesbergweg 10-14, 55099 Mainz, Germany

## Abstract

The complete molecule of the title compound, C_24_H_24_N_6_O_2_, is generated by a crystallographic inversion centre. The benzotriazole rings form dihedral angles of 2.10 (7)° with the central aromatic ring. The crystal packing is consolidated by π–π inter­actions, with centroid–centroid distances of 3.6234 (10) Å, together with weak C—H⋯π inter­actions.

## Related literature

For the biological activity of *N*-oxide and benzotriazole derivatives see: Katarzyna *et al.*(2005 ▶); Sarala *et al.* (2007 ▶). For applications of benzotriazole, see: Kopec *et al.* (2008 ▶); Krawczyk & Gdaniec (2005 ▶); Smith *et al.* (2001 ▶); Sha *et al.* (1996 ▶). For 1-hydroxy­benzotriazole, see: Anderson *et al.* (1963 ▶); Bosch *et al.* (1983 ▶).
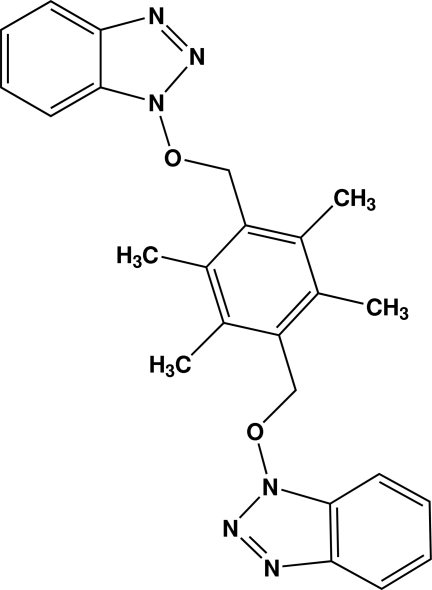


## Experimental

### 

#### Crystal data

C_24_H_24_N_6_O_2_
*M* *_r_* = 428.49
Monoclinic, 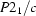

*a* = 9.3895 (6) Å
*b* = 7.5960 (2) Å
*c* = 15.7471 (13) Å
β = 110.770 (3)°
*V* = 1050.13 (11) Å^3^
*Z* = 2
Cu *K*α radiation
μ = 0.73 mm^−1^
*T* = 193 K
0.51 × 0.26 × 0.19 mm

#### Data collection

Enraf–Nonius CAD-4 diffractometer
Absorption correction: ψ scan (*CORINC*; Draeger & Gattow, 1971 ▶) *T*_min_ = 0.707, *T*_max_ = 0.873
2075 measured reflections
1996 independent reflections
1905 reflections with *I* > 2σ(*I*)
*R*_int_ = 0.026
3 standard reflections frequency: 60 min intensity decay: 1%

#### Refinement

*R*[*F* ^2^ > 2σ(*F* ^2^)] = 0.052
*wR*(*F* ^2^) = 0.147
*S* = 1.10
1996 reflections
147 parameters
H-atom parameters constrained
Δρ_max_ = 0.32 e Å^−3^
Δρ_min_ = −0.33 e Å^−3^

### 

Data collection: *CAD-4 EXPRESS* (Enraf–Nonius, 1994 ▶); cell refinement: *CAD-4 EXPRESS*; data reduction: *CORINC* (Draeger & Gattow, 1971 ▶); program(s) used to solve structure: *SHELXS97* (Sheldrick, 2008 ▶); program(s) used to refine structure: *SHELXL97* (Sheldrick, 2008 ▶); molecular graphics: *SHELXTL* (Sheldrick, 2008 ▶); software used to prepare material for publication: *SHELXTL* and *PLATON* (Spek, 2009 ▶).

## Supplementary Material

Crystal structure: contains datablocks global, I. DOI: 10.1107/S1600536809010782/bt2912sup1.cif

Structure factors: contains datablocks I. DOI: 10.1107/S1600536809010782/bt2912Isup2.hkl

Additional supplementary materials:  crystallographic information; 3D view; checkCIF report

## Figures and Tables

**Table 1:** Hydrogen-bond geometry (Å, °)

*D*—H⋯*A*	*D*—H	H⋯*A*	*D*⋯*A*	*D*—H⋯*A*
C6—H6⋯*Cg*2^i^	0.95	2.82	3.700 (2)	154

Symmetry code: (i) 
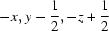
. *Cg*2 is the centroid of the C4–C9 ring.

**Fig. 1.**
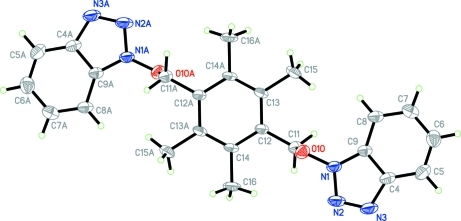
The molecular structure of the title compound, showing 50% probability displacement ellipsoids and the atom numbering scheme [symmetry code: -x + 1,-y + 1,-z + 1].

**Fig. 2.**
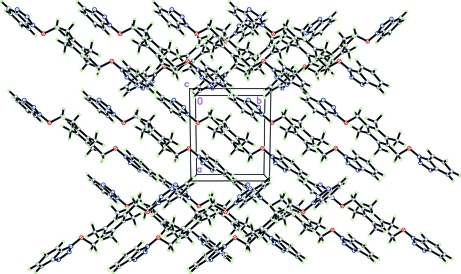
The crystal packing of the title compound, viewed down the c axis.

## supplementary crystallographic information

### Comment

Benzotriazole derivatives show biological activities such as anti-inflammatory, diuretic, antiviral and antihypertensive agents (Katarzyna *et al.*, 2005; Sarala *et al.*, 2007). It is used as a corrosion inhibitor, antifreeze agent, ultraviolet light stabilizer for plastics and as an antifoggant in photography (Krawczyk & Gdaniec, 2005; Smith *et al.*, 2001). *N*-aryloxy derivatives of benzotriazoles have antimycobacterial activity (Kopec *et al.*, 2008). Benzotriazole possessing three vicinal N atoms, is used as an antifouling and antiwear reagent (Sha *et al.*, 1996). 1-Hydroxybenzotriazole is widely being used as a reagent for peptide synthesis (Anderson *et al.*, 1963). The crystal structure of benzotriazole 1-oxide has been reported (Bosch *et al.*, 1983). Due to the above mentioned applications of benzotriazole we have synthesized and report here the crystal structure of the title compound (I).

The asymmetric unit of (I) comprises of half molecule of the title compound (Fig. 1), the other half is symmetry generated [symmetry code: 1 - *x*,1 - *y*,1 - *z*].   The benzotriazole ring is essentially planar with the maximum deviation from planarity being 0.015 (18) Å for atom C8. The mean plane of the benzotriazole rings (N1—N3/C4—C9; N1A—N3A/C4A—C9A) forming a dihedral angles of 2.10 (7)° and 2.09 (7)° respectively, with the phenyl ring (*C*12 - *C*14/C12A-C14A), indicating that all the three are almost coplanar.

The crystal packing (Fig.2) is stabilized by π—π stacking interactions [*Cg*2—*Cg*3^i^= 3.6234 (10) Å; *Cg*2: (C4—C9); *Cg*3:(C12—C14/C12A—C14A): Symmetry code: (i) *x*, -1 + *y*, *z*]; [*Cg*2—*Cg*3^ii^= 3.6234 (10) Å; *Cg*2: (C4—C9); *Cg*3:(C12—C14/C12A—C14A): Symmetry code: (ii) 1 - *x*, -*y*,1 - *z*] together with weak C—H···π interactions. (Fig.2).

### Experimental

A mixture of 1,4-bis(bromomethyl)-2,3,5,6-tetramethyl-benzene (0.320 g, 1 mmol) and sodium salt of 1-hydroxybenzotriazole (0.314 2 mmol) in ethanol (10 ml) was heated at 333 K with stirring for 30 min. The product formed was filtered off and dried. The product was dissolved in ethanol and on slow evaporation crystals suitable for x-ray diffraction are obtained.

### Refinement

All the H atoms were positioned geometrically (C_aromatic_—H=0.95 Å, C_methyl_—H=0.98  or C_methylene_—H=0.99 Å)  and refined using a riding model with, *U*_iso_(H)=1.2U_eq_(C) and 1.5U_eq_(C_methyl_). A rotating group model was used for the methyl groups.

### Crystal data

**Table:**

C_24_H_24_N_6_O_2_	*F*(000) = 452
*M**_r_* = 428.49	*D*_x_ = 1.355 Mg m^−^^3^
Monoclinic, *P*2_1_/*c*	Cu *K*α radiation, λ = 1.54178 Å
Hall symbol: -P 2ybc	Cell parameters from 25 reflections
*a* = 9.3895 (6) Å	θ = 65–70°
*b* = 7.5960 (2) Å	µ = 0.73 mm^−^^1^
*c* = 15.7471 (13) Å	*T* = 193 K
β = 110.770 (3)°	Block, colourless
*V* = 1050.13 (11) Å^3^	0.51 × 0.26 × 0.19 mm
*Z* = 2	

### Data collection

**Table:**

Enraf–Nonius CAD-4 diffractometer	1905 reflections with *I* > 2σ(*I*)
Radiation source: rotating anode	*R*_int_ = 0.026
graphite	θ_max_ = 70.0°, θ_min_ = 5.0°
ω/2θ scans	*h* = −11→10
Absorption correction: ψ scan (CORINC; Draeger & Gattow, 1971)	*k* = 0→9
*T*_min_ = 0.707, *T*_max_ = 0.873	*l* = 0→19
2075 measured reflections	3 standard reflections every 60 min
1996 independent reflections	intensity decay: 1%

### Refinement

**Table:**

Refinement on *F*^2^	Primary atom site location: structure-invariant direct methods
Least-squares matrix: full	Secondary atom site location: difference Fourier map
*R*[*F*^2^ > 2σ(*F*^2^)] = 0.052	Hydrogen site location: inferred from neighbouring sites
*wR*(*F*^2^) = 0.147	H-atom parameters constrained
*S* = 1.10	*w* = 1/[σ^2^(*F*_o_^2^) + (0.0832*P*)^2^ + 0.5*P*] where *P* = (*F*_o_^2^ + 2*F*_c_^2^)/3
1996 reflections	(Δ/σ)_max_ < 0.001
147 parameters	Δρ_max_ = 0.32 e Å^−^^3^
0 restraints	Δρ_min_ = −0.33 e Å^−^^3^

### Special details

**Table:**

Geometry. All esds (except the esd in the dihedral angle between two l.s. planes) are estimated using the full covariance matrix. The cell esds are taken into account individually in the estimation of esds in distances, angles and torsion angles; correlations between esds in cell parameters are only used when they are defined by crystal symmetry. An approximate (isotropic) treatment of cell esds is used for estimating esds involving l.s. planes.
Refinement. Refinement of F^2^ against ALL reflections. The weighted R-factor wR and goodness of fit S are based on F^2^, conventional R-factors R are based on F, with F set to zero for negative F^2^. The threshold expression of F^2^ > 2sigma(F^2^) is used only for calculating R-factors(gt) etc. and is not relevant to the choice of reflections for refinement. R-factors based on F^2^ are statistically about twice as large as those based on F, and R- factors based on ALL data will be even larger.

### Fractional atomic coordinates and isotropic or equivalent isotropic displacement parameters (Å^2^)

**Table:**

	*x*	*y*	*z*	*U*_iso_*/*U*_eq_	
N1	0.25022 (16)	−0.07614 (18)	0.47932 (9)	0.0302 (3)	
N2	0.17398 (19)	−0.1201 (2)	0.53429 (10)	0.0385 (4)	
N3	0.08837 (19)	−0.2555 (2)	0.49841 (11)	0.0408 (4)	
C4	0.10897 (19)	−0.2985 (2)	0.41828 (12)	0.0317 (4)	
C5	0.0438 (2)	−0.4308 (2)	0.35473 (14)	0.0396 (4)	
H5	−0.0276	−0.5118	0.3627	0.047*	
C6	0.0873 (2)	−0.4389 (3)	0.28015 (13)	0.0408 (5)	
H6	0.0438	−0.5264	0.2353	0.049*	
C7	0.1948 (2)	−0.3209 (2)	0.26842 (12)	0.0380 (4)	
H7	0.2212	−0.3309	0.2157	0.046*	
C8	0.26238 (19)	−0.1922 (2)	0.33068 (12)	0.0321 (4)	
H8	0.3367	−0.1146	0.3235	0.039*	
C9	0.21514 (17)	−0.1820 (2)	0.40554 (11)	0.0270 (4)	
O10	0.34983 (13)	0.06259 (15)	0.50086 (8)	0.0317 (3)	
C11	0.27184 (18)	0.2246 (2)	0.45748 (11)	0.0296 (4)	
H11A	0.1907	0.2561	0.4812	0.036*	
H11B	0.2253	0.2087	0.3909	0.036*	
C12	0.39164 (17)	0.3661 (2)	0.48003 (10)	0.0248 (4)	
C13	0.47071 (18)	0.3997 (2)	0.42060 (10)	0.0259 (4)	
C14	0.42305 (17)	0.4639 (2)	0.55983 (10)	0.0255 (4)	
C15	0.4428 (2)	0.2893 (2)	0.33658 (12)	0.0381 (4)	
H15A	0.3797	0.3554	0.2830	0.057*	
H15B	0.3901	0.1806	0.3416	0.057*	
H15C	0.5404	0.2601	0.3305	0.057*	
C16	0.3446 (2)	0.4206 (3)	0.62620 (13)	0.0397 (5)	
H16A	0.4183	0.4283	0.6884	0.060*	
H16B	0.3032	0.3009	0.6147	0.060*	
H16C	0.2616	0.5044	0.6185	0.060*	

### Atomic displacement parameters (Å^2^)

**Table:**

	*U*^11^	*U*^22^	*U*^33^	*U*^12^	*U*^13^	*U*^23^
N1	0.0380 (7)	0.0273 (7)	0.0337 (7)	−0.0030 (6)	0.0229 (6)	0.0022 (5)
N2	0.0543 (9)	0.0351 (8)	0.0403 (8)	−0.0001 (7)	0.0344 (7)	0.0050 (6)
N3	0.0532 (9)	0.0338 (8)	0.0513 (9)	−0.0041 (7)	0.0383 (8)	0.0039 (7)
C4	0.0330 (8)	0.0285 (8)	0.0417 (9)	0.0010 (6)	0.0231 (7)	0.0057 (7)
C5	0.0331 (9)	0.0320 (9)	0.0582 (12)	−0.0044 (7)	0.0219 (8)	−0.0012 (8)
C6	0.0381 (10)	0.0375 (10)	0.0450 (10)	0.0005 (7)	0.0124 (8)	−0.0077 (8)
C7	0.0462 (10)	0.0399 (10)	0.0335 (9)	0.0059 (8)	0.0208 (8)	0.0008 (7)
C8	0.0380 (9)	0.0308 (9)	0.0360 (9)	0.0012 (7)	0.0234 (7)	0.0045 (7)
C9	0.0304 (8)	0.0251 (8)	0.0304 (8)	0.0025 (6)	0.0170 (6)	0.0044 (6)
O10	0.0343 (6)	0.0260 (6)	0.0382 (7)	−0.0022 (5)	0.0170 (5)	0.0013 (5)
C11	0.0316 (8)	0.0272 (8)	0.0344 (8)	0.0003 (6)	0.0171 (7)	0.0021 (6)
C12	0.0304 (7)	0.0243 (8)	0.0253 (7)	0.0010 (6)	0.0170 (6)	0.0016 (6)
C13	0.0354 (8)	0.0266 (8)	0.0223 (7)	0.0033 (6)	0.0184 (6)	0.0001 (6)
C14	0.0325 (8)	0.0284 (8)	0.0241 (7)	0.0040 (6)	0.0204 (6)	0.0037 (6)
C15	0.0540 (11)	0.0389 (10)	0.0312 (9)	−0.0041 (8)	0.0272 (8)	−0.0091 (7)
C16	0.0553 (11)	0.0421 (10)	0.0386 (9)	−0.0027 (8)	0.0375 (9)	0.0024 (8)

### Geometric parameters (Å, °)

**Table:**

N1—N2	1.3465 (18)	C11—C12	1.504 (2)
N1—C9	1.354 (2)	C11—H11A	0.9900
N1—O10	1.3694 (17)	C11—H11B	0.9900
N2—N3	1.304 (2)	C12—C14	1.399 (2)
N3—C4	1.382 (2)	C12—C13	1.409 (2)
C4—C5	1.397 (3)	C13—C14^i^	1.394 (2)
C4—C9	1.400 (2)	C13—C15	1.509 (2)
C5—C6	1.375 (3)	C14—C13^i^	1.394 (2)
C5—H5	0.9500	C14—C16	1.5129 (19)
C6—C7	1.410 (3)	C15—H15A	0.9800
C6—H6	0.9500	C15—H15B	0.9800
C7—C8	1.370 (3)	C15—H15C	0.9800
C7—H7	0.9500	C16—H16A	0.9800
C8—C9	1.401 (2)	C16—H16B	0.9800
C8—H8	0.9500	C16—H16C	0.9800
O10—C11	1.4707 (19)		
			
N2—N1—C9	112.41 (14)	C12—C11—H11A	110.5
N2—N1—O10	120.24 (13)	O10—C11—H11B	110.5
C9—N1—O10	127.34 (13)	C12—C11—H11B	110.5
N3—N2—N1	107.67 (14)	H11A—C11—H11B	108.7
N2—N3—C4	108.54 (14)	C14—C12—C13	120.49 (15)
N3—C4—C5	131.05 (16)	C14—C12—C11	119.57 (14)
N3—C4—C9	108.58 (15)	C13—C12—C11	119.94 (14)
C5—C4—C9	120.37 (16)	C14^i^—C13—C12	119.49 (14)
C6—C5—C4	117.17 (16)	C14^i^—C13—C15	119.67 (14)
C6—C5—H5	121.4	C12—C13—C15	120.84 (15)
C4—C5—H5	121.4	C13^i^—C14—C12	119.98 (13)
C5—C6—C7	121.79 (17)	C13^i^—C14—C16	119.62 (14)
C5—C6—H6	119.1	C12—C14—C16	120.38 (15)
C7—C6—H6	119.1	C13—C15—H15A	109.5
C8—C7—C6	122.08 (16)	C13—C15—H15B	109.5
C8—C7—H7	119.0	H15A—C15—H15B	109.5
C6—C7—H7	119.0	C13—C15—H15C	109.5
C7—C8—C9	115.90 (15)	H15A—C15—H15C	109.5
C7—C8—H8	122.1	H15B—C15—H15C	109.5
C9—C8—H8	122.1	C14—C16—H16A	109.5
N1—C9—C4	102.80 (14)	C14—C16—H16B	109.5
N1—C9—C8	134.53 (15)	H16A—C16—H16B	109.5
C4—C9—C8	122.67 (16)	C14—C16—H16C	109.5
N1—O10—C11	110.19 (11)	H16A—C16—H16C	109.5
O10—C11—C12	106.29 (12)	H16B—C16—H16C	109.5
O10—C11—H11A	110.5		
			
C9—N1—N2—N3	0.3 (2)	C5—C4—C9—C8	−0.7 (3)
O10—N1—N2—N3	−179.78 (14)	C7—C8—C9—N1	−178.85 (17)
N1—N2—N3—C4	−0.5 (2)	C7—C8—C9—C4	1.9 (2)
N2—N3—C4—C5	−179.59 (18)	N2—N1—O10—C11	−92.95 (16)
N2—N3—C4—C9	0.5 (2)	C9—N1—O10—C11	86.90 (18)
N3—C4—C5—C6	179.40 (18)	N1—O10—C11—C12	−176.52 (11)
C9—C4—C5—C6	−0.7 (3)	O10—C11—C12—C14	−88.45 (17)
C4—C5—C6—C7	0.9 (3)	O10—C11—C12—C13	91.90 (16)
C5—C6—C7—C8	0.3 (3)	C14—C12—C13—C14^i^	−2.3 (3)
C6—C7—C8—C9	−1.6 (3)	C11—C12—C13—C14^i^	177.30 (13)
N2—N1—C9—C4	−0.03 (18)	C14—C12—C13—C15	177.14 (15)
O10—N1—C9—C4	−179.88 (14)	C11—C12—C13—C15	−3.2 (2)
N2—N1—C9—C8	−179.42 (18)	C13—C12—C14—C13^i^	2.4 (3)
O10—N1—C9—C8	0.7 (3)	C11—C12—C14—C13^i^	−177.29 (13)
N3—C4—C9—N1	−0.29 (18)	C13—C12—C14—C16	−176.44 (15)
C5—C4—C9—N1	179.80 (15)	C11—C12—C14—C16	3.9 (2)
N3—C4—C9—C8	179.20 (15)		

Symmetry codes: (i) −*x*+1, −*y*+1, −*z*+1.

### Hydrogen-bond geometry (Å, °)

**Table:**

*D*—H···*A*	*D*—H	H···*A*	*D*···*A*	*D*—H···*A*
C6—H6···Cg2^ii^	0.95	2.82	3.700 (2)	154

Symmetry codes: (ii) −*x*, *y*−1/2, −*z*+1/2.
